# Differential roles of hypoxia and innate immunity in juvenile and adult dermatomyositis

**DOI:** 10.1186/s40478-016-0308-5

**Published:** 2016-04-27

**Authors:** Corinna Preuße, Yves Allenbach, Olaf Hoffmann, Hans-Hilmar Goebel, Debora Pehl, Josefine Radke, Alexandra Doeser, Udo Schneider, Rieke H.E. Alten, Tilmann Kallinich, Olivier Benveniste, Arpad von Moers, Benedikt Schoser, Ulrike Schara, Werner Stenzel

**Affiliations:** Department of Neuropathology, Charité - Universitätsmedizin Berlin, Charité Campus Mitte, Charitéplatz 1, D-10117 Berlin, Germany; Department of Internal Medicine and Clinical Immunology, Pitié-Salpêtrière University Hospital, Paris, France; Department of Neurology, St. Josefs Hospital, Potsdam, Germany; Department of Neuropathology, University Medicine, Johannes Gutenberg University, Mainz, Germany; Department of Rheumatology, Charité - Universitätsmedizin Berlin, Berlin, Germany; Department of Internal Medicine II, Schlossparkklinik, Berlin, Germany; Department of Pediatrics, Charité - Universitätsmedizin Berlin, Berlin, Germany; Department of Pediatrics and Neuropediatrics, DRK Klinikum Westend, Berlin, Germany; Friedrich-Baur-Institut, Ludwig Maximilians Universität, Munich, Germany; Department of Neuropediatrics, Universitätsklinikum, Essen, Germany

**Keywords:** Dermatomyositis, Perifascicular atrophy, Hypoxia, Macrophages, Inflammation, Type-I interferon

## Abstract

Dermatomyositis (DM) can occur in both adults and juveniles with considerable clinical differences. The links between immune-mediated mechanisms and vasculopathy with respect to development of perifascicular pathology are incompletely understood. We investigated skeletal muscle from newly diagnosed, treatment-naïve juvenile (jDM) and adult dermatomyositis (aDM) patients focusing on hypoxia-related pathomechanisms, vessel pathology, and immune mechanisms especially in the perifascicular region. Therefore, we assessed the skeletal muscle biopsies from 21 aDM, and 15 jDM patients by immunohistochemistry and electron microscopy. Transcriptional analyses of genes involved in hypoxia, as well as in innate and adaptive immunity were performed by quantitative Polymerase chain reaction (qPCR) of whole tissue cross sections including perifascicular muscle fibers.

Through these analysis, we found that basic features of DM, like perifascicular atrophy and inflammatory infiltrates, were present at similar levels in jDM and aDM patients. However, jDM was characterized by predominantly hypoxia-driven pathology in perifascicular small fibers and by macrophages expressing markers of hypoxia. A more pronounced regional loss of capillaries, but no relevant activation of type-1 Interferon (IFN)-associated pathways was noted. Conversely, in aDM, IFN-related genes were expressed at significantly elevated levels, and Interferon-stimulated gene (ISG)15 was strongly positive in small perifascicular fibers whereas hypoxia-related mechanisms did not play a significant role.

In our study we could provide new molecular data suggesting a conspicuous pathophysiological ‘dichotomy’ between jDM and aDM: In jDM, perifascicular atrophy is tightly linked to hypoxia-related pathology, and poorly to innate immunity. In aDM, perifascicular atrophy is prominently associated with molecules driving innate immunity, while hypoxia-related mechanisms seem to be less relevant.

**Electronic supplementary material:**

The online version of this article (doi:10.1186/s40478-016-0308-5) contains supplementary material, which is available to authorized users.

## Introduction

Dermatomyositis (DM) is a rare and severe inflammatory disease, which predominantly involves skeletal muscle and skin but may also affect internal organs, e.g. coronary arteries and heart. Juvenile DM (jDM), defined by an age of onset below 16 years, and adult dermatomyositis (aDM) display considerable differences with respect to clinical features and typical complications [37]. For instance, jDM often follows a chronic course with relative resistance to therapy and an increased risk of permanent damage characterized by skeletal muscle atrophy or calcinosis [15, 31]. Yet the overall prognosis of aDM may actually be worse due to a substantially increased risk of malignancy not present in jDM [23]. Intestinal involvement is frequent in jDM and rare in aDM, while associated interstitial lung disease is much more frequently observed in aDM. Hence, the clinical spectra of jDM and aDM are clearly distinct, but our understanding of any underlying differences in pathophysiology is hampered by a lack of studies directly comparing adults and children with DM (3).

On the morphological level, presence of inflammatory infiltrates predominantly in the perimysium, as well as ischemia resulting from ‘vasculopathy’ and pathognomonic perifascicular muscle fiber atrophy, are recognized hallmarks of the disease [26, 29]. However, the connotation of each of these features and their interrelation are not well characterized and remain controversial [15]. Adaptive and innate immune mechanisms involving interferon-associated molecules appear to mediate endothelial tubuloreticular formations and, according to more recent results, perifascicular atrophy [5, 9, 12, 36]. Dendritic cells rather than macrophages have been identified as a source of type I interferons and were deemed to play a central role in DM pathogenesis, specifically in the development of perifascicular atrophy [32]. Adaptive immune mechanisms including expression of major histocompatibility complex class I (MHC I) [19, 22] and activation of T and B cells [20, 30, 33] are additional features of DM. Apart from these immune-mediated effects, hypoxia-related pathology due to vascular changes has been recognized as an important mechanism. Using 3D reconstructions, Gitiaux and coworkers have recently shown that inflammation in larger arcade arteries may be more relevant to hypoxia-related pathology than capillary loss [10]. They illustrated that perifascicular atrophy in DM is probably the consequence of a certain inflammatory vasculopathy, which is not a vasculitis *sensu stricto*, and follows a characteristic pattern of capillary dropout.

In this study we aimed to compare the relative contribution of hypoxia-related vs. inflammatory pathology in jDM and aDM by analyzing the expression of hypoxia- and IFN-associated genes, as well as functional macrophage activation in perifascicular and centrally located muscle fibers.

## Materials and Methods

### Patients and biopsy specimens

We analyzed cryopreserved skeletal muscle biopsy specimens from 36 patients fulfilling the clinico-pathological criteria of aDM (*n* = 21) or jDM (*n* = 15) and control biopsy specimens (*n* = 10) [14, 39, 40]. Criteria for inclusion were typical symptoms like muscle weakness, muscle pain, skin rash/Gottron’s papules and dysphagia. In addition the presence of undulating tubules in endothelia of all skeletal muscle biopsy specimens was demonstrated. Myositis-specific antibodies (MSAs), and myositis-associated antibodies (MAAs) in the serum of patients were inconstantly measured and are reported if available retroactively. Patients with atypical histopathological findings were excluded, e.g. patients with excessive macrophagic inflammation like IMAMs or patients with other overlapping diseases (overlap syndromes). The available clinical information is given in Table 1.

**Table 1:** Clinical and paraclinical data as well as therapeutic details for jDM and aDM patients

Age (mean ± SD)		juvenile (*n* = 15)	adult (*n* = 21)	NC (*n* = 10)
		8 ^+^/_−_4 years	57 ^+^/_−_15 years	7 ^+^/_−_6 years and 45 ^+^/_−_11 years
Gender	female	47 % (7)	67 % (14)	30 % (3)
	male	53 % (8)	33 % (7)	70 % (7)
Biopsy location	M. deltoideus		57 % (12)	50 % (5)
	M. quadriceps	87 % (13)	5 % (1)	
	unknown	13 % (2)	38 % (8)	50 % (5)
CK	normal	20 % (3)	14 % (3)	100 % (10)
	>1-fold	60 % (9)	29 % (6)	
	>10-fold	13 % (2)	38 % (8)	
	unknown	7 % (1)	19 % (4)	
Symptoms	typical skin rash	73 % (11)	52 % (11)	
	dysphagia	20 % (3)	10 % (2)	20 % (2)^a^
	muscle weakness	73 % (11)	67 % (14)	20 % (2)^a^
	muscle pain	60 % (9)	52 % (11)	50 % (5)^a^
	others	27 % (4)	38 % (8)	
	unknown	13 % (2)	5 % (1)	
Duration of	in months	5 ^+^/_−_4 (11)	3 ^+^/_−_3 (10)	7 ^+^/_−_1 (4)
symptoms	>1 years	7 % (1)	5 % (1)	20 % (2)
	unknown	20 % (3)	48 % (10)	19 % (4)
Tubuloreticular formations (EM)	positive	53 % (8)	76 % (16)	n.d.
	unknown	47 % (7)	24 % (5)	
MSAs	positive	7 % (1)	19 % (4)	n.d.
	negative	67 % (10)	33 % (7)	
	unknown	26 % (4)	48 % (10)	
Clinical outcome	remission	53 % (8)	29 % (6)	
	improvement	20 % (3)	10 % (2)	
	remitting-relapsing	27 % (4)	10 % (2)	
	no improvement	0 % (0)	5 % (1)	
	unknown	0 % (0)	48 % (10)	100 % (10)

^a^subjective complaints which could not be substantiated by clinical or ancillary exams, *n.d*. not done

Control biopsies comprised five juvenile and five adult patients with non-specific muscular complaints. Control patients had normal clinical findings, normal serum creatine kinase, no laboratory evidence of systemic inflammation, negative results for MSAs, and MAAs (as tested at time of biopsy), and no morphological abnormalities in the biopsy tissue. For the analysis, all healthy control ‘patients’ results were pooled into one control group, since expression levels of the analyzed markers did not differ between the age groups. All biopsies were obtained prior to any treatment. Each biopsy consisted of a single block of tissue, and the entire cross-section of each sample was analyzed at multiple levels. All specimens were cryopreserved after removal at − 80 °C prior to diagnostic work-up. Informed consent was obtained from all patients at each institution. The study was reviewed and approved by the institutional ethics board of the Charité (No. EA1/204/11).

Patients were tested for MSA and MAA with different commercially available standard kits using immunoblot technologies. These tests included various antibodies such as Jo1, Mi-2, SRP, PL7, PL12, Ro52 or KU.

### Histology, enzyme histochemistry, and immunohistochemistry

7 μm cryostat sections were stained by various routine preparations including H&E, Gömöri trichrome, alkaline phosphatase, COX-SDH (adapted staining protocol from Dubowitz et al. [7] staining overnight, followed by 2 h SDH staining at RT), MHC class I (DAKO, Glostrup, Denmark, clone W6/32 1:100), MHC class II (DAKO, M0775 1:100), CD56/anti-neural cell adhesion molecule (NCAM, (clone ERIC-1, AbD Serotec, Kidlington, United Kingdom 1:100), (neonatal myosin heavy chain (MHCneo) (Leica, Wetzlar, Germany, clone WBMHCn 1:100), HIF-1α predominantly staining myofibers (Abnova, Taipeh, Taiwan, clone H1alpha67, 1:40), HIF-1α predominantly staining macrophages (Abcam, Cambridge, UK, clone EP12154, 1:100), VEGFA (CellSignaling, Danvers MA, USA, 1:100), CD4 (Novocastra, Newcastle, GB, clone 4B12, 1:20), CD8 (DAKO, clone C8/144B, 1:100), CD68 (DAKO, clone EBM11, 1:100), CD79a (Epitomics, Burlingame, CA, USA, clone EP82, 1:200), CD303/BDCA2 (Millipore, Schwalbach, Germany, clone 10E6.1, 1:50), CD206 (Acris, Herford, Germany, clone AM05589PU-N, 1:500), CD31 (DAKO, clone M0823, 1:25), ISG15 (Abcam, clone ab14374, 1:50), laminin α5 (Millipore, clone MAB1924, 1:30.000) and laminin α2 (Chemicon, MAB1922Z, 1:5000) are shown. Staining was performed, using the *i*view-Ventana DAB (diaminobenzidine)-Detection Kit (Ventana, Tucson, Arizona, 85755 USA), or the (3-amino-9-ethylcarbazole) AEC-Detection Kit (Ventana). Appropriate biotinylated secondary antibodies were used, and visualization of the reaction product was carried out on a Benchmark XT immunostainer (Ventana) in a standardized manner. Omission of primary antibodies in control sections resulted in absence of any cellular labeling and demonstrated specificity of the primary antibody. Also, appropriate positive and negative controls (tissue reaction) were used where necessary. In addition we used normal muscle tissue as negative control (or physiological control e.g. staining of arterioles by c5b9, MHC class I positivity of capillaries) for all reactions. Double immunostaining was performed using AEC and DAB as substrates after serial incubation with laminin α5 and CD206 antibodies.

Stains and immunostaining for fluorescent markers were performed in staining chambers after fixation in acetone for 10 min. The sections were then blocked with the appropriate serum (1:10 in PBS) dependent upon the source of the secondary antibody, and incubated with the afore-mentioned primary antibodies over-night at 4 °C or for 1 h at RT. After a washing step, the secondary antibody was added for 1 h. For double immunostaining, with the purpose to show co-localization of two cellular structures, the above-mentioned protocol was performed using the first primary antibody and afterwards the same protocol was repeated with the second primary antibody and appropriate secondary antibodies. After a final washing step, the sections were aqueously mounted and stored at 4 °C.

### VAS score for severity

To evaluate overall affection of different muscle compartments, we used a modified VAS (visual analog scale) score described by Wedderburn et.al. [40]. We scored the defined four different compartments individually (inflammatory, vascular, muscular and connective tissue) depending on overall affection (see Additional 1: Figure S1a), to highlight the variable affection of these compartments:

score 0 = no abnormality, score 10 = most abnormal.

All values are presented as box-whiskers blots min-max with means and standard deviation.

### Semi-quantitative score for ISG15, HIF1α and VEGF

To evaluate expression of ISG15 (Interferon-stimulated gene 15), HIF1α (Hypoxia-inducible factor 1-alpha) and VEGF (Vascular Endothelial Growth Factor) in muscle specimens of DM patients, a semi quantitative evaluation was applied to immune histological stainings in jDM and aDM patients biopsies (see Additional 1: Figure S1b). The quantification was performed in a blinded manner by a trained pathologist.

score 0 = no staining; 1 = single positive myofibres; 2 = 1–2 layers in most fascicles; 3 = multiple layers in most fascicles; 4 = multiple layers in most fascicles and centrofascicular staining.

All values are presented as Box-Whiskers blot min-max with means and standard deviation.

### Quantitative real-time polymerase chain reaction (qPCR)

RNA was extracted from whole tissue using the trizol/chloroform method, according to the manufacturer’s instruction (Invitrogen, Carlsbad, CA, USA). Thereafter, RNA was resuspended and the concentration of total RNA was photometrically determined with a TECAN fluorescence plate reader (Tecan, Männedorf, Switzerland). The RNA was reverse transcribed using the High-Capacity cDNA Archive Kit (Applied Biosystems, Foster City, CA), according to the manufacturer’s protocol, by using 2 μg of total RNA per sample [28]. For qPCR reactions, 2 ng of cDNA were used. All genes were run as triplicates and each run contained the reference gene (*PGK1*) as internal control. The expression (meaning CT value) of this reference gene was comparable in all analyzed samples, including healthy controls, and unaffected by duration of the disease. To exclude loading differences and variations between different runs all target genes were normalized to expression of *PGK1*. For analysis, the 7900HT Fast Real-Time PCR System (Applied Biosystems, Foster City, CA) was used, running conditions: 95 °C 0:20, 95 °C 0:01, 60 °C 0:20, 45 cycles (values above 40 cycles were defined as not expressed).

The qPCR assay identification numbers are as follows:

*IFNG*: Hs00989291_m1; *IFNA*: Hs00265051_s1; *STAT1*: Hs01013989_m1; *STAT2*: Hs01013123_m1; *STAT3*: Hs00374280_m1; *STAT6*: Hs00598625_m1; *TNFA*: Hs00174128_m1; *FBXO32*: Hs01041408_m1; *cox2*: Hs00153133_m1; *RORG*: Hs01076122_m1; *IL1B*: Hs01555410_m1; *IL4*: Hs00929862_m1; *IL5*: Hs01548712_g1; *IL6*: Hs00985639_m1; *IL12B*: Hs01011518_m1; *IL13*: Hs99999038_m1; *IL17*: Hs00174383_m1; *IL21*: Hs00222327_m1; *IL27*: Hs00377366_m1; *ISG15*: Hs01921425_s1; *OAS3*: Hs00196324_m1, *MX1*: Hs00895608_m1; *RIPK*: Hs00169407_m1; *MDA5*: Hs01070332_m1; *CCL17*: Hs00171074_m1; *MRC1* (CD206): Hs00267207_m1; *TGFB*: Hs00998133_m1; *VEGFA*: Hs00900055_m1; *HIF1A*: Hs00153153_m1; *MIF*: Hs00236988_g1; *PGK*: Hs99999906_m1.

Data are either presented as logarithmic fold-change (logRQ; compared to non-diseased control patients’ biopsies, calculation 2^-ddCt) or as dCT in case of TH1, TH2 and TH17 marker, since some of the markers are not expressed in healthy controls and calculation of the RQ value is therefore not possible. All values are presented as Box-Whiskers blot min-max with means and standard deviation.

### Capillary/fiber ratio

Capillaries and muscle fibers were manually counted on laminin α5-stained sections using the ImageJ software. Ten high power fields (HPF) of 0.16 mm^2^ each were evaluated per biopsy, and the ratio of capillaries to fibers per case was calculated. Since the capillary to fiber ratio between juveniles and adults in control biopsies did not differ, all cases were all used as a single control group.

### Statistics

Kruskal-Wallis one-way ANOVA was applied to analyze quantitative differences of mRNA transcripts, using Bonferroni correction of the post hoc tests. This was done to compare all groups of patients, not assuming normalization of the data. It was not possible to perform statistics with the Kruskal-Wallis test for the expression of *IL12B*, *IL4*, *IL5*, *IL13*, *IL17* and *IL21*, because the respective cytokines were not expressed at detectable levels in normal controls. The level of significance was set at *p* < 0.05. For VAS and ISG15 scores the Mann–Whitney *U* test was used. Statistics were calculated with the GraphPad Prism 5.02 software (GraphPad Software, Inc., La Jolla, California, USA).

## Results

### Analysis of clinical data

Twenty-one patients with dermatomyositis were adult and 15 patients were juvenile (Table 1). In the juvenile group the female to male ratio was approximately 50:50, while in the aDM group the ratio was 70:30. This result matched data from the literature, indicating an increased proportion of female patients in aDM [41].

Juvenile DM patients tended to present more severe (Table 1) and varied symptoms than aDM patients. Therapeutic strategies varied individually, but glucocorticoid treatment was the most common therapy, intravenous immunoglobulins (IvIG) were given only occasionally, and both, methotrexate (MTX) or azathioprine (AZT) were used as long-term immunosuppressants. Therapeutic outcome was also inconstant. It has to be noted, that both adult and juvenile patients who were diagnosed early in the disease process and treated with corticosteroids and methotrexate (MTX) or azathioprine (AZT) had a good prognosis. However as this was a retroactive evaluation, information about therapeutic success was not available for many of the adult DM patients, limiting the interpretation of this set of data. One juvenile patient had a poor outcome with wheelchair dependency, and one jDM patient developed calcinosis of the skeletal muscles. Neoplasms were identified in two aDM patients during follow-up. Antibodies were studied prior to biopsy in 60 % of aDM and 50 % of jDM patients.

### Inflammatory infiltration is similar in jDM and aDM patients, while capillary loss is more pronounced in jDM

**Fig. 1:**
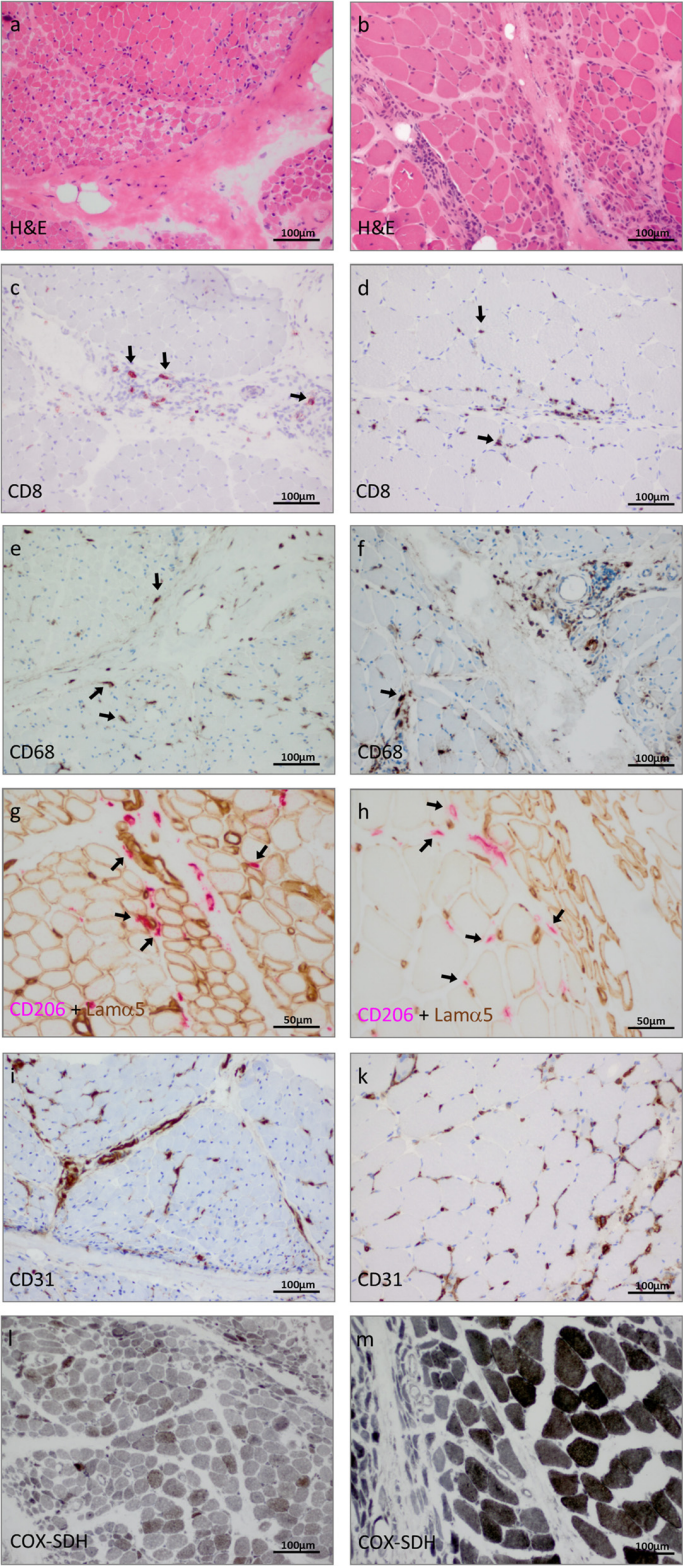
Histological analysis of jDM (a, c, e, g, i, l), and aDM (b, d, f, h, k, m) muscle biopsy specimens. Multiple layers of atrophic myofibers in perifascicular regions are illustrated both in jDM (**a**) and in aDM (**b**) by H&E and by MHCI staining in jDM (inlay a) as well as in aDM (inlay b). CD8^+^ lymphocytic infiltrates, are localized in the perimysium, and extend focally into the endomysium both in jDM and in aDM (**c**, **d**). CD68^+^ macrophages were identified both in jDM and in aDM (**e**, **f**). Close proximity between CD206^+^ macrophages (AEC, red) with laminin a5^+^ capillaries (DAB, brown) in the perifascicular region in jDM (**g**), which is less obvious in aDM (**h**). Capillary dropout is more evident in jDM as compared to aDM as illustrated by CD31 staining (**i**, **k**). COX-SDH enzymo-histochenmical preparations show severe pallor by COX staining giving rise to a greyish appearance in jDM (**l**), while the identical staining procedure done in parallel produced this aspect only in the perifascicular atrophic region in aDM patients (**m**). Arrows highlighting the respective features in photomicrographs

**Fig. 2:**
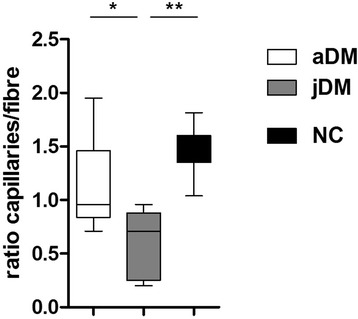
Capillary fiber ratio in jDM vs. aDM. Ratio of capillaries vs. myofibers is significantly lower in jDM as compared to aDM and healthy controls; jDM *n* = 7; aDM *n* = 13; NC *n* = 7

To characterize inflammatory infiltrates in patients with dermatomyositis we employed various histological stains. As shown in Fig. 1 exemplarily, the overall morphological pattern with perifascicular atrophy (Fig. 1a, b), and distribution of inflammatory leukocytes (CD4^+^ (not shown) and CD8^+^ cells (Fig. 1c, d) were similar in both groups of patients. Relatively few CD79a^+^ B cells were also detected in the endomysium and the perimysium (data not shown). In addition, also expression of MHC class I molecules was found predominantly in perifascicular regions (Additional 2: Figure S2a, b). Throughout the biopsies, the most prevalent population of inflammatory cells were CD68^+^ macrophages (Fig. 1e, f) with a conspicuous proportion of CD206^+^ cells. Macrophages accumulated predominantly in the perifascicular region, extending into the endomysium (Fig. 1g, h). In addition, CD206^+^ macrophages (red) were regularly identified in close proximity to laminin α5^+^ capillaries (brown) in close proximity to laminin α5^+^ atrophic fibers (sarcolemmal staining; brown) in jDM-, but less conspicuously in aDM patients (Fig. 1g, h). Capillary loss was more prominent in juvenile patients, frequently occurring also in the center of fascicles, as identified by Lamα5 and CD31 staining (Fig. 1g, k). Pallor of COX staining [25] in COX-SDH enzyme histochemical reactions was striking in jDM and comprised whole fascicles, whereas it was confined to the perifascicular region only in aDM patients indicating more pronounced oxidative stress in jDM patients’ skeletal muscles (Fig. 1l, m). Assessment of the capillary to fiber ratio was significantly reduced in jDM patients as compared to aDM patients and healthy controls (Fig. 2).

Electronmicroscopic analysis of endomysial capillaries revealed tubuloreticular formations in all adult and juvenile patients muscle biopsy specimens. In addition, only single CD303^+^ plasmacytoid dendritic cells (pDCs) were identified with equally low abundance in jDM and aDM (Additional 2: Figure S2c–e).

### Perifascicular atrophic fibers are associated with hypoxia in jDM, while they are associated with IFN-associated molecules in aDM

**Fig. 3:**
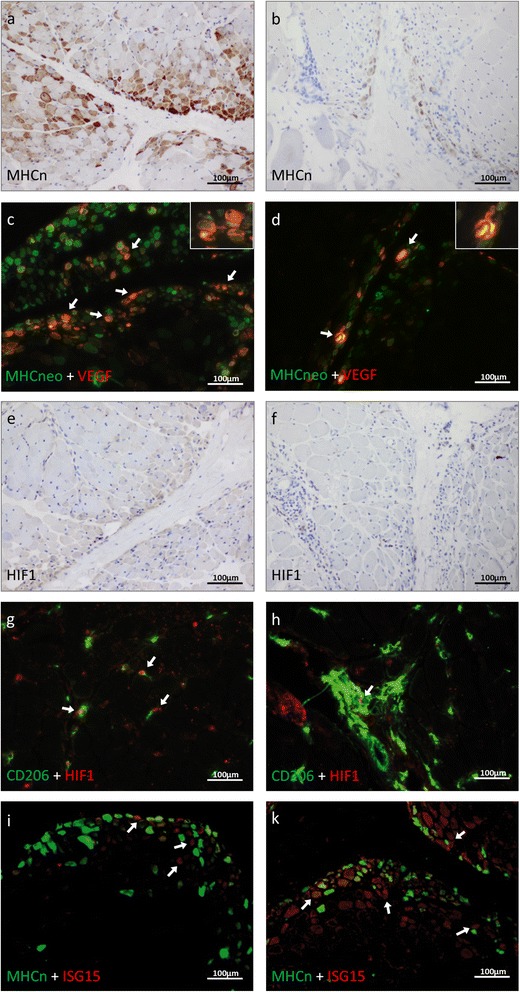
*Role of hypoxia and IFN1-associated molecules in perifascicular regions of skeletal muscle biopsies derived from jDM and aDM patients.* CD56^+^ myofibers are more prominent and more widespread in the perifascicular region in jDM (**a**) compared to aDM (**b**). Neonatal myosin + myofibers (green) frequently co-stain with VEGF^+^ (red) myofibers in jDM (**c**), and only occasional double positive fibers are detected in aDM samples (**d**; original magnification x200). Numerous HIF1a^+^ myofibers are detected in the perifascicular region in jDM (**e**), while this is not the case in aDM (**f**). Using a different clone highlights double positive HIF1a^+^ (red) and CD206^+^ macrophages (green) in jDM (**g**), but CD206^+^ macrophages do not co-label with HIF1a in aDM (**h**). ISG15 (red) is co-expressed in a few perifascicular atrophic neonatal myosin^+^ myofibers (green) in jDM (**i**), but ISG15 is much more widespread and strongly stains multiple layers of the perifascicular regions in aDM and co-stains with neonatal myosin (**k**)

Perifascicular atrophy as defined by consensus criteria for a severity score in jDM [39, 40] was evaluated. Visualization of different modalities was performed by staining with CD56 (Fig. 3a; jDM, b; aDM) and neonatal myosin (Fig. 3c, i green in jDM; and d, k green in aDM), as well as MHC class I (Fig. 1a, b insert), or MHC class II (data not shown), where various amounts of atrophic, immature and regenerating fibers adjacent to the perimysium, generally more pronounced in jDM patients biopsies, as compared to aDM could be detected. Among these small/atrophic neonatal myosin^+^ fibers (green), numerous ones co-stained with markers of hypoxia such as VEGFA [16] (red) (Fig. 3c). In jDM biopsy samples myofibers were also HIF1α-positive in jDM biopsy samples (Fig. 3e). On the contrary, aDM patients’ biopsies only showed exceptional co-staining of neonatal myosin with VEGFA (Fig. 3d), HIF1α^+^ fibers were rare or absent (Fig. 3f). Using a different HIF1α antibody (clone 12154), which stains macrophages rather than myofibers, double-immunofluorescence further illustrates CD206^+^ macrophages (green) co-expressing HIF1α (red) in jDM (Fig. 3g). As also shown in Fig. 1h, CD206^+^ macrophages were present in aDM muscle as well, but they did not co-stain with HIF1α (Fig. 3h).

Conversely, ISG15 immunoreactivity (red) of perifascicular small/atrophic neonatal myosin^+^ fibers (green) was only exceptionally detected in jDM (Fig. 3i), whereas in aDM, perifascicular small/atrophic neonatal myosin^+^ fibers (green) exhibited co-staining of ISG15 and neonatal myosin and showed conspicuous immune reactivity with ISG15 (red) (Fig. 3k). Notably, ISG15 immunoreactivity was not confined to atrophic fibers in aDM but involved numerous neonatal myosin–negative, non-atrophic muscle fibers in and adjacent to the perifascicular region. A semi quantitative score of the ISG15 staining is also shown in Additional file 1: Figure S1.

### Hypoxia-related molecular pathways are activated in jDM, whereas expression of IFN1-associated genes is more relevant in aDM

**Fig. 4:**
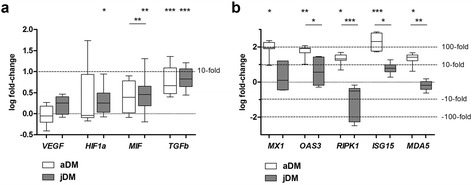
Transcription of relevant genes associated with the IFN1 response as well as with hypoxia. Gene expression of *HIF1a and MIF* is significantly higher in jDM vs. control skeletal muscle tissue, while these markers are not significantly up-regulated in aDM vs. controls. Expression of *TGFb* however is significantly up-regulated in both groups (**a**). Gene expression of *ISG15, MX1, OAS3, MDA5* and *RIPK* is significantly higher in aDM as compared to normal control muscle tissue, while conversely, these genes are not expressed at a significantly different level in jDM as compared to controls (**b**). Of note is that the expression of *ISG15* and *MX1* is also highly variable between individual aDM patients. Data are represented as Box-Whiskers blot min-max with mean and standard deviation, level of significance *p* < 0.05; jDM *n* = 10; aDM *n* = 7; NC *n* = 10

In whole tissue homogenates, qPCR showed significantly increased expression of the hypoxia-related genes *HIF1A* and *MIF* in jDM patients compared to controls, whereas no significant up-regulation was detected in aDM skeletal muscle tissue (Fig. 4a). In contrast, *TGFb* was significantly elevated in jDM and aDM patients’ skeletal muscle. *VEGFA* expression did not reach statistical significance in both groups (as compared to controls) although a trend for upregulation could be noted in jDM patients (Fig. 4a). Both *VEGFA-* and *HIF1A* expression did not reach statistically significant difference between jDM and aDM patients.

Since ISG15 immunoreactivity was extensively detected on muscle fibers of the perifascicular area in aDM patients’ samples, the expression levels of the IFN1-associated genes *MX1, OAS3, RIPK, ISG15* and *MDA5* [32, 36] were measured. Expression of *MX1, OAS3, ISG15*, *RIPK* and *MDA5* were significantly increased in aDM patients’ skeletal muscle biopsies compared to controls and except for *MX1*, also when compared to jDM samples. In contrast, jDM patients showed no significantly up-regulated expression of these IFN1-associated genes in the skeletal muscle as compared to controls (Fig. 4b), corresponding to the absent or low ISG15 immunoreactivity in jDM biopsies.

**Fig. 5:**
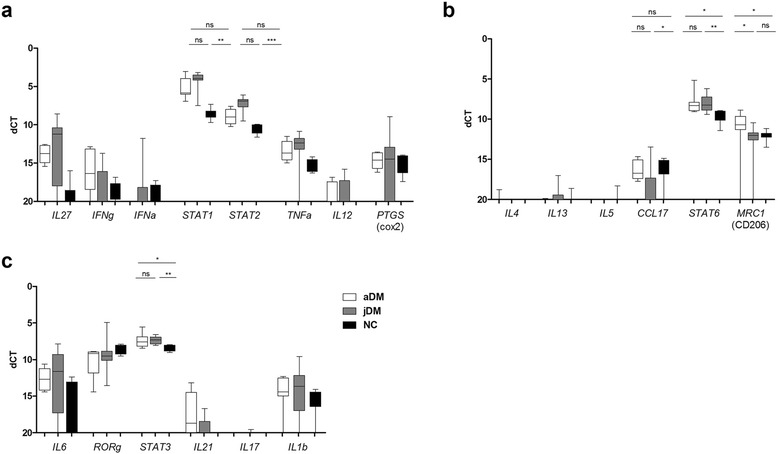
Expression of pro- and anti-inflammatory markers in DM patients. Cytokines driving Th1-, Th2- and Th17 pathways were analyzed in adult and juvenile patients. Expression levels of STAT1 and STAT2 were significantly increased in jDM patients, while STAT6 showed a significant increase in aDM muscle biopsies. Overall Th17-associated immune markers were expressed on a very low level. Data are represented as Box-Whiskers blot min-max with mean and standard deviation, level of significance *p* < 0.05; jDM *n* = 10; aDM *n* = 15; NC *n* = 10

Cytokines driving Th1-, Th2- and Th17 pathways were additionally analyzed (Fig. 5). Expression levels of the Th1-associated genes *STAT1* and *STAT2* were significantly increased in jDM patients (Fig. 5a). *STAT6*, a signal transducer and activator of transcription factor of Th2-immunity, e.g. activating macrophages to express the mannose receptor, was significantly increased in aDM muscle compared to jDM and controls (Fig. 5b). As a marker involved in Th17 immunity, we found a non-significant trend for up-regulation of *IL6* in jDM, and a significant increase of *STAT3* in jDM and aDM (Fig. 5c). Very low expression of *IL21* or *IL17* indicated that Th17-associated immune responses were not operative at a relevant level.

## Discussion

The present study identifies significant differences between jDM and aDM regarding the presence of hypoxic and type-1 interferon-associated markers: (i) In jDM, but significantly less relevant in aDM, hypoxia-associated changes are detectable in the perifascicular region. (ii) Small/atrophic fibers, which express markers of hypoxia in jDM, also express various markers of regeneration and immaturity, suggesting a link between perifascicular atrophy and hypoxia. (iii) CD206^+^ macrophages co-express HIF-1α in jDM but not in aDM, indicating an involvement of macrophages in pathways of hypoxia in jDM. In addition, these macrophages are frequently found in close proximity to capillaries in jDM but not in aDM. (iv) Capillary dropout is more pronounced in jDM muscle biopsy specimens compared to aDM muscle biopsy specimens. (v) A significant proportion of atrophic fibers of aDM patients are immuno-positive for ISG15, while ISG15 immunoreactivity is relatively less in jDM. IFN1-associated genes are expressed in aDM samples but not conspicuously in jDM.

Consistent with our findings, type-I interferon-inducible changes and activation of the innate immune system have previously been described in aDM. *ISG15* and myxovirus resistance gene A (*MXA*) were up-regulated in aDM, and ISG15 was detected in the perifascicular region of aDM muscle biopsies [32]. ISG15 staining was reported to co-localize with CD31^+^ capillaries in aDM, while PM and sIBM patients’ biopsies were ISG15-negative (17). Moreover, immunoreactivity of perifascicular muscle fibers for RIG-1, an intracellular innate immune receptor, was reported as a specific finding in aDM [11, 36]. An interesting mechanism of autocrine self-sustained immune activation was hypothesized [11]. In jDM, up-regulation of IFN-induced transcripts was reported in skin biopsies of four patients compared to those in Duchenne muscular dystrophy or healthy controls [34], but a comparison to aDM patients was not performed. We expand these data by demonstrating the absence of significant gene expression of *ISG15*, *MDA5*, *MX1*, *RIPK*, and *OAS3* as well as low protein staining of ISG15 in jDM patients, while confirming the reported presence and regional distribution of ISG15 staining in aDM. Further, levels of gene expression and staining-intensity of ISG15 paralleled nicely in jDM and aDM. Causes and effects of IFN-related changes in DM are incompletely understood. Virus-induced autoimmunity has been hypothesized [38], but viruses have not been detected in skeletal muscle of patients with Idiopathic inflammatory myopathies (IIMs) so far [18]. An important question relates to whether the IFN signature is a non-specific effect of the presence of inflammatory cells, e.g. pDCs [12], or whether it is specifically induced by the myofiber pathology of DM. The low number of pDCs in skeletal muscle biopsies of both our groups of patients did not show further quantitative differences, which prompts us to hypothesize that IFN1-associated molecules are rather expressed by myofibers than by pDCs in dermatomyositis. It is also unclear whether and how IFNs are responsible for the specific pattern of perifascicular skeletal myofiber atrophy (PMA). PMA is a myopathological diagnostic hallmark of DM [14] but absent in other inflammatory myopathies - e.g. sporadic inclusion body myositis, immune-mediated necrotizing myopathy, overlap myositis or nonspecific myositis – even when large numbers of atrophic and/or regenerating myofibers are present.

As a possible link between inflammation and PMA, an inflammatory vasculopathy has been described in DM, and hypothesized to cause rarefaction or progressive dropout of capillaries and hypoxic or ischemic reactions of the tissue, possibly involving complement-mediated destruction of capillaries and subsequent hypoperfusion or microinfarcts [6, 17, 21]. The causal role of a primary inflammatory vasculopathy at the capillary level was, however, challenged recently, arguing that complement deposition in capillaries is not consistently present in DM, whereas necrotizing myopathy with pipestem capillaries is not associated with PMA despite a very prominent complement deposition [8]. PMA is also not observed in livedoid vasculopathy which also features dramatic capillary dropout and perimysial inflammation in muscle tissue [1]. In summary, inflammation *per se* and capillary dropout may also not fully explain DM-specific PMA.

We have addressed this issue in a comparative way by analyzing PMA by different methods. Indeed, it seems intriguing that both mechanisms the hypoxia-related and the IFN-related one should lead to PMA.

In juveniles with DM, the co-localization of VEGFA, and neonatal myosin^+^ (MHCneo) small/atrophic fibers as well as the HIF-1α positive atrophic fibers suggest a relevant role of hypoxia-associated mechanisms driving PMA. Consistently, we found increased expression of hypoxia-associated genes on the RNA level in the respective tissue of biopsy samples. Using a microdissection technique, a severely reduced titin protein content was reported as a potential mechanism of PMA whereas a causal role of hypoxia or ischemia in DM was questioned [32]. Our results indicate that capillary dropout may be linked to presence of CD206^+^HIF-1α^+^ macrophages, which were frequently detectable in direct proximity to capillaries predominantly in jDM. Hence angiogenesis has been directly linked to VEGF/HIF signaling [16], with macrophages polarized by HIF molecules playing a significant role [2, 3]. Increased transforming growth factor beta (*TGFb)* expression, which can induce HIF-1α and contribute to expression of CD206 in macrophages, fits well in our observations in jDM muscle specimens and may also lead to HIF-1α expression on atrophic muscle fibers. *TGFb*−expression is not specific for hypoxia though and can regulate fibrosis and numerous other biological pathways both during development and in adulthood [24] as well; therefore, it is not contradictory to find elevated expression of this molecule in aDM as well. Prolonged hypoxia has the propensity to up-regulate HIF-1α in macrophages in vitro [35], however, these authors show a decreased expression of CD40 and CD206, which is contrary to what we found in vivo. Thus, we think that CD206^+^HIF1α^+^ macrophages may contribute to hypoxic damage of capillaries especially in jDM.

In depth analysis of capillary and vascular pathology in jDM and aDM patients revealed that avascular perimysium correlated with perifascicular atrophy, while vascular perimysium did not [26]. Pestronk et al. underscore the relevance of a tight anatomic relation between capillary pathology and distribution of fiber atrophy, and the likeliness of a hypoxic aetiopathology in DM in general [25]. A note of caution has to be mentioned since the capillary density between children and adults, as well as in different muscles from individual patients may differ.

Of note, one study analyzing HIF-1α, HIF-1β and HIF-2α as well as VEGF and VEGF-R by immunohistochemistry found HIF-1α expression on endothelial cells while VEGF was also detected on muscle fibers [13, 29]. The authors describe a pattern of expression of HIF-2α as well as VEGF-R in perifascicular atrophic fibers and argue that this could be due to an adaptive mechanism attempting to restore blood supply [13, 29]. Attention was also given to the phenomenon that early and late phases as well as phases of active disease may show differences in VEGF expression. These authors argue for an involvement of VEGF in hypoxia regeneration and angiogenesis, with similar findings in PM and DM and no explanation with respect to the specific development of PMA [13]. Our data obtained on the mRNA level show, that *HIF1a* and *MIF* are significantly increased in jDM patients, as compared to controls, but unfortunately we were not able to illustrate significant differences between jDM, and aDM patients. Thus, our PCR data do not strongly underscore our conclusions i.e. that jDM patients show more- hypoxia-associated alterations than aDM patients. These data however do not undermine our conclusions as well, since a trend was seen in all measurements, which strongly argues that the differential expression of these proteins, seen in the perifascicular regions by immunofluorescence was somehow ‘diluted’ in the whole muscle lysates used for qPCR. In theory, it could also be possible, that the hypoxia-associated mechanisms are especially strong in specific subgroups of jDM patients. This remains to be determined in futures studies, using larger series of well-selected patients e.g. with defined auto-antibody profiles. In theory HIF1a, VEGF and MIF might also be active in hypoxia-independent mechanisms, which remain to be elucidated.

Pronounced hypoxia in jDM skeletal muscle specimens may also, at least partially, explain the fact that calcinosis in general occurs in juvenile DM patients but only rarely in adults. Calcinosis has indeed been associated with continuous hypoperfusion in the myocardium and in a mouse model [4, 27], and mitochondrial dysfunction, a feature which is very prominent in DM and specifically in jDM [25], as we confirm here. The clear predominance of IFN-related markers in aDM in contrast to markers of hypoxia in jDM argues that both phenomena may actually not be directly linked to each other. It is therefore intriguing how two distinct mechanisms should result in the same specific pattern of atrophy, PMA, in aDM and jDM.

## Conclusion

In conclusion, mechanisms linked to a defective IFN pathway are more prevalent in aDM, and hypoxia is less relevant indicating that both pathways are probably independent from each other. This also argues against the hypothesis that one phenomenon is the cause of the other one. Hence, the IFN-associated pathology is most likely not the consequence of hypoxia, and *vice versa*, as we show in our study.

### Consent

Informed consent was obtained from all individual participants included in this study.

## Additional files

Additional file 1:
**Figure S1A.** *VAS and score of ISG15, HIF1*α *and VEGF in DM patients.* “Severity” of tissue affection in the vascular and muscle compartment as measured by the modified VAS score was more pronounced in jDM compared to aDM patients, while the inflammatory and connective tissue compartments scored on the same level (a); jDM *n* = 11; aDM *n* = 8. Using the semi-quantitative score revealed that aDM patients were significantly stronger affected by ISG15 expression when compared to jDM patients (b). On the other hand jDM patients showed more extensive staining for HIF1α and VEGF (b); jDM *n* = 10–13; aDM *n* = 10–14. (JPG 300 kb) Additional file 2:
**Figure S2A.** Staining with MHCI and abundance of CD303^+^ plasmacytoid dendritic cells. Staining with MHCI demonstrates a perifascicular pattern in jDM (a), as well as aDM (b) patients. Staining for CD303^+^ plasmacytoid dendritic cells (pDCs) revealed only low numbers by histology in aDM (c) and jDM (d) and was confirmed by cell count (e); jDM *n* = 5; aDM *n* = 8. (TIF 2112 kb)

## References

[1] Allenbach Y, Tourte M, Stenzel W, Goebel HH, Maisonobe T, Frances C, Barete S, Benveniste O (2015). Expanding the spectrum of livedoid vasculopathy: peculiar neuromuscular manifestations. Neuropathol Appl Neurobiol.

[2] Bosco MC, Delfino S, Ferlito F, Puppo M, Gregorio A, Gambini C, Gattorno M, Martini A, Varesio L (2009). The hypoxic synovial environment regulates expression of vascular endothelial growth factor and osteopontin in juvenile idiopathic arthritis. J Rheumatol.

[3] Bosco MC, Puppo M, Blengio F, Fraone T, Cappello P, Giovarelli M, Varesio L (2008). Monocytes and dendritic cells in a hypoxic environment: Spotlights on chemotaxis and migration. Immunobiology.

[4] Buschmann CT, Stenzel W, Martin H, Heppner FL, Guddat SS, Tsokos M (2013). Calcified myocardial necrosis in pediatric patients after cardiopulmonary resuscitation. Forensic Sci Med Pathol.

[5] Cappelletti C, Baggi F, Zolezzi F, Biancolini D, Beretta O, Severa M, Coccia EM, Confalonieri P, Morandi L, Mora M, Mantegazza R, Bernasconi P (2011). Type I interferon and Toll-like receptor expression characterizes inflammatory myopathies. Neurology.

[6] Dalakas MC (1998). Molecular immunology and genetics of inflammatory muscle diseases. Arch Neurol.

[7] Dubowitz V, Sewry C, Oldfors A (2013). Muscle Biopsy.

[8] Emslie-Smith AM, Engel AG (1991). Necrotizing myopathy with pipestem capillaries, microvascular deposition of the complement membrane attack complex (MAC), and minimal cellular infiltration. Neurology.

[9] Feldman D, Hoar RM, Niemann WH, Valentine T, Cukierski M, Hendrickx AG (1986). Tubuloreticular inclusions in placental chorionic villi of rhesus monkeys after maternal treatment with interferon. Am J Obstet Gynecol.

[10] Gitiaux C, Kostallari E, Lafuste P, Authier FJ, Christov C, Gherardi RK (2014). Whole microvascular unit deletions in dermatomyositis. Ann Rheum Dis.

[11] Greenberg SA (2014). Sustained autoimmune mechanisms in dermatomyositis. J Pathol.

[12] Greenberg SA, Pinkus JL, Pinkus GS, Burleson T, Sanoudou D, Tawil R, Barohn RJ, Saperstein DS, Briemberg HR, Ericsson M, Park P, Amato AA (2005). Interferon-alpha/beta-mediated innate immune mechanisms in dermatomyositis. Ann Neurol.

[13] Grundtman C, Tham E, Ulfgren AK, Lundberg IE (2008). Vascular endothelial growth factor is highly expressed in muscle tissue of patients with polymyositis and patients with dermatomyositis. Arthritis Rheum.

[14] Hoogendijk JE, Amato AA, Lecky BR, Choy EH, Lundberg IE, Rose MR, Vencovsky J, de Visser M, Hughes RA (2004). 119th ENMC international workshop: trial design in adult idiopathic inflammatory myopathies, with the exception of inclusion body myositis, 10–12 October 2003, Naarden, The Netherlands. Neuromuscul Disord.

[15] Khanna S, Reed AM (2010). Immunopathogenesis of juvenile dermatomyositis. Muscle Nerve.

[16] Kim YW, Byzova TV (2014). Oxidative stress in angiogenesis and vascular disease. Blood.

[17] Kissel JT, Mendell JR, Rammohan KW (1986). Microvascular deposition of complement membrane attack complex in dermatomyositis. N Engl J Med.

[18] Leff RL, Love LA, Miller FW, Greenberg SJ, Klein EA, Dalakas MC, Plotz PH (1992). Viruses in idiopathic inflammatory myopathies: absence of candidate viral genomes in muscle. Lancet.

[19] Li CK, Varsani H, Holton JL, Gao B, Woo P, Wedderburn LR (2004). MHC Class I overexpression on muscles in early juvenile dermatomyositis. J Rheumatol.

[20] Lopez De Padilla CM, Vallejo AN, Lacomis D, McNallan K, Reed AM (2009). Extranodal lymphoid microstructures in inflamed muscle and disease severity of new-onset juvenile dermatomyositis. Arthritis Rheum.

[21] Miles L, Bove KE, Lovell D, Wargula JC, Bukulmez H, Shao M, Salisbury S, Bean JA (2007). Predictability of the clinical course of juvenile dermatomyositis based on initial muscle biopsy: a retrospective study of 72 patients. Arthritis Rheum.

[22] Nagaraju K, Raben N, Loeffler L, Parker T, Rochon PJ, Lee E, Danning C, Wada R, Thompson C, Bahtiyar G, Craft J, Hooft Van Huijsduijnen R, Plotz P (2000). Conditional up-regulation of MHC class I in skeletal muscle leads to self-sustaining autoimmune myositis and myositis-specific autoantibodies. Proc Natl Acad Sci U S A.

[23] Olazagasti JM, Baez PJ, Wetter DA, Ernste FC (2015). Cancer risk in dermatomyositis: a meta-analysis of cohort studies. Am J Clin Dermatol.

[24] Padgett RW, Reiss M (2007). TGFbeta superfamily signaling: notes from the desert. Development.

[25] Pestronk A (2011). Acquired immune and inflammatory myopathies: pathologic classification. Curr Opin Rheumatol.

[26] Pestronk A, Schmidt RE, Choksi R (2010). Vascular pathology in dermatomyositis and anatomic relations to myopathology. Muscle Nerve.

[27] Pipinos II, Swanson SA, Zhu Z, Nella AA, Weiss DJ, Gutti TL, McComb RD, Baxter BT, Lynch TG, Casale GP (2008). Chronically ischemic mouse skeletal muscle exhibits myopathy in association with mitochondrial dysfunction and oxidative damage. Am J Physiol Regul Integr Comp Physiol.

[28] Preusse C, Goebel HH, Held J, Wengert O, Scheibe F, Irlbacher K, Koch A, Heppner FL, Stenzel W (2012). Immune-mediated necrotizing myopathy is characterized by a specific Th1-m1 polarized immune profile. Am J Pathol.

[29] Probst-Cousin S, Neundorfer B, Heuss D (2010). Microvasculopathic neuromuscular diseases: lessons from hypoxia-inducible factors. Neuromuscul Disord.

[30] Radke J, Pehl D, Aronica E, Schonenberg-Meinema D, Schneider U, Heppner FL, de Visser M, Goebel HH, Stenzel W (2014). The lymphoid follicle variant of dermatomyositis. Neurol Neuroimmunol Neuroinflamm.

[31] Rider LG, Katz JD, Jones OY (2010). Developments in the classification and treatment of the juvenile idiopathic inflammatory myopathies. Rheum Dis Clin North Am.

[32] Salajegheh M, Kong SW, Pinkus JL, Walsh RJ, Liao A, Nazareno R, Amato AA, Krastins B, Morehouse C, Higgs BW, Jallal B, Yao Y, Sarracino DA, Parker KC, Greenberg SA (2010). Interferon-stimulated gene 15 (ISG15) conjugates proteins in dermatomyositis muscle with perifascicular atrophy. Ann Neurol.

[33] Salajegheh M, Pinkus JL, Amato AA, Morehouse C, Jallal B, Yao Y, Greenberg SA (2010). Permissive environment for B-cell maturation in myositis muscle in the absence of B-cell follicles. Muscle Nerve.

[34] Shrestha S, Wershil B, Sarwark JF, Niewold TB, Philipp T, Pachman LM (2010). Lesional and nonlesional skin from patients with untreated juvenile dermatomyositis displays increased numbers of mast cells and mature plasmacytoid dendritic cells. Arthritis Rheum.

[35] Staples KJ, Sotoodehnejadnematalahi F, Pearson H, Frankenberger M, Francescut L, Ziegler-Heitbrock L, Burke B (2011). Monocyte-derived macrophages matured under prolonged hypoxia transcriptionally up-regulate HIF-1alpha mRNA. Immunobiology.

[36] Suarez-Calvet X, Gallardo E, Nogales-Gadea G, Querol L, Navas M, Diaz-Manera J, Rojas-Garcia R, Illa I (2014). Altered RIG-I/DDX58-mediated innate immunity in dermatomyositis. J Pathol.

[37] Tansley SL, McHugh NJ, Wedderburn LR (2013). Adult and juvenile dermatomyositis: are the distinct clinical features explained by our current understanding of serological subgroups and pathogenic mechanisms?. Arthritis Res Ther.

[38] Tezak Z, Hoffman EP, Lutz JL, Fedczyna TO, Stephan D, Bremer EG, Krasnoselska-Riz I, Kumar A, Pachman LM (2002). Gene expression profiling in DQA1*0501+ children with untreated dermatomyositis: a novel model of pathogenesis. J Immunol.

[39] Varsani H, Charman SC, Li CK, Marie SK, Amato AA, Banwell B, Bove KE, Corse AM, Emslie-Smith AM, Jacques TS, Lundberg IE, Minetti C, Nennesmo I, Rushing EJ, Sallum AM, Sewry C, Pilkington CA, Holton JL, Wedderburn LR (2015). Validation of a score tool for measurement of histological severity in juvenile dermatomyositis and association with clinical severity of disease. Ann Rheum Dis.

[40] Wedderburn LR, Varsani H, Li CK, Newton KR, Amato AA, Banwell B, Bove KE, Corse AM, Emslie-Smith A, Harding B, Hoogendijk J, Lundberg IE, Marie S, Minetti C, Nennesmo I, Rushing EJ, Sewry C, Charman SC, Pilkington CA, Holton JL (2007). International consensus on a proposed score system for muscle biopsy evaluation in patients with juvenile dermatomyositis: a tool for potential use in clinical trials. Arthritis Rheum.

[41] Zouagui A, Abourazzak S, Idrissi ML, Souilmi FZ, Chaouki S, Atmani S, Bouharrou A, Hida M (2011). Actuality of juvenile dermatomyositis. Joint Bone Spine.

