# Supplementary figures and images for: Differential roles of hypoxia and innate immunity in juvenile and adult dermatomyositis
Source: Acta Neuropathol Commun. 2016 Apr 27;4:45. doi: 10.1186/s40478-016-0308-5 (PMC4847347; doi:10.1186/s40478-016-0308-5)

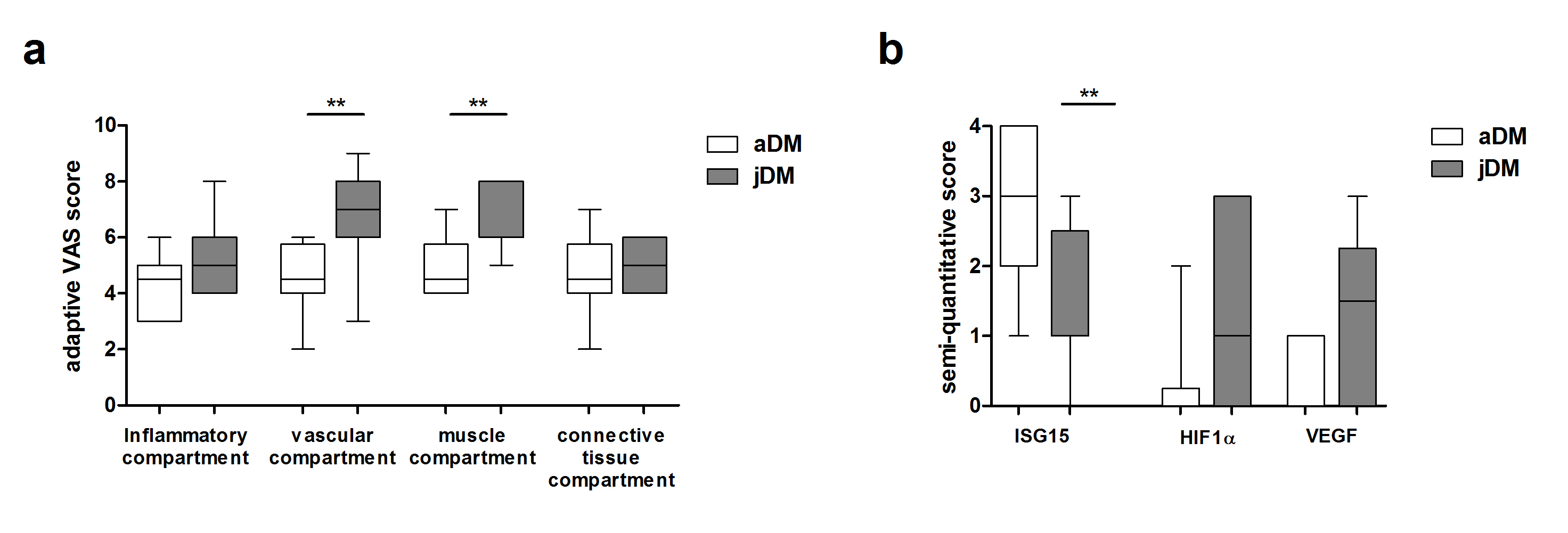

Supplement: Additional file 1: — Figure S1A. VAS and score of ISG15, HIF1α and VEGF in DM patients. “Severity” of tissue affection in the vascular and muscle compartment as measured by the modified VAS score was more pronounced in jDM compared to aDM patients, while the inflammatory and connective tissue compartments scored on the same level (a); jDM n = 11; aDM n = 8. Using the semi-quantitative score revealed that aDM patients were significantly stronger affected by ISG15 expression when compared to jDM patients (b). On the other hand jDM patients showed more extensive staining for HIF1α and VEGF (b); jDM n = 10–13; aDM n = 10–14. (JPG 300 kb) [file 40478_2016_308_MOESM1_ESM.jpg]

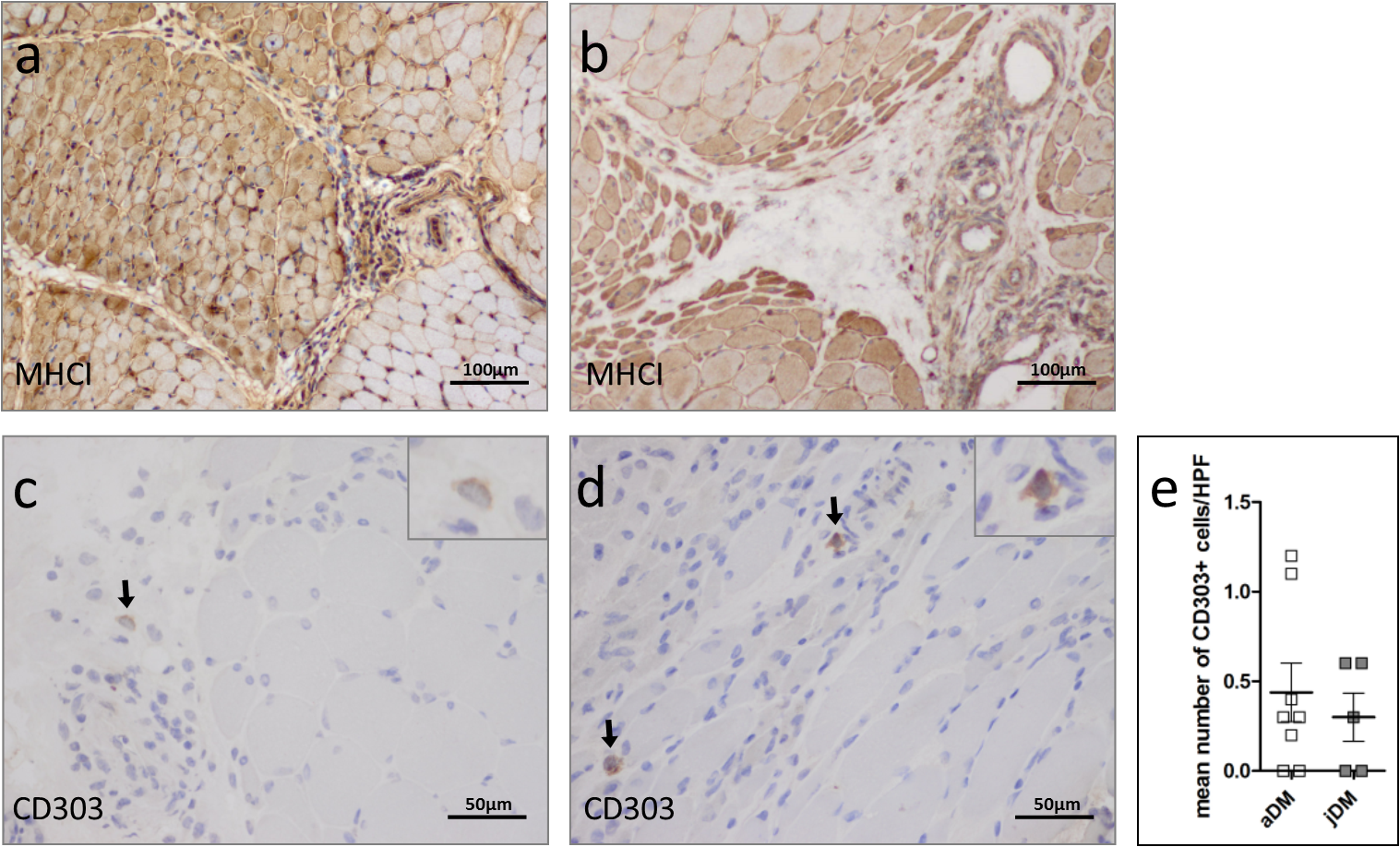

Supplement: Additional file 2: — Figure S2A. Staining with MHCI and abundance of CD303+ plasmacytoid dendritic cells. Staining with MHCI demonstrates a perifascicular pattern in jDM (a), as well as aDM (b) patients. Staining for CD303+ plasmacytoid dendritic cells (pDCs) revealed only low numbers by histology in aDM (c) and jDM (d) and was confirmed by cell count (e); jDM n = 5; aDM n = 8. (TIF 2112 kb) [file 40478_2016_308_MOESM2_ESM.tif]
